# Surgical management of a 6-month-old infant with bilateral intramural total anomalous coronary artery origin from the pulmonary artery

**DOI:** 10.1016/j.xjtc.2026.102330

**Published:** 2026-03-20

**Authors:** Alexander Moiroux-Sahraoui, Régis Gaudin, Diala Khraiche, Ségolène Bernheim, Sophie Malekzadeh-Milani, Alessia Callegari, Damien Bonnet, Olivier Raisky, Neil Derridj

**Affiliations:** a Department of Pediatric Cardiac Surgery, Hôpital Necker Enfants Malades, AP-HP, Paris, France; b Université Paris Cité, Paris, France; c Department of Pediatric Cardiology, Hôpital Necker Enfants Malades, AP-HP, Paris, France

**Figure:**
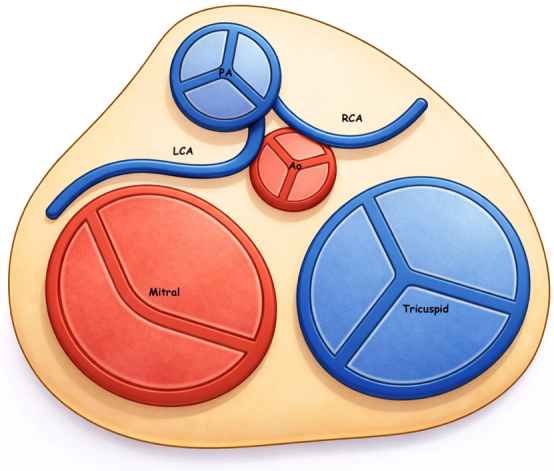
TCAPA with bilateral intramural course.

**Central Message**

We present a unique case of a 6-month-old infant who survived bilateral intramural TCAPA. The successful surgical strategy involved unroofing of both vessels.

Congenital coronary artery anomalies affect an estimated 0.17 to 1.2% of the population.^1^ Although most variants are clinically silent, they collectively represent the second-leading cause of sudden cardiac death in children.^2^ The most lethal subgroup involves coronary vessels originating from the pulmonary artery (PA). The best-known variant, anomalous left coronary artery from the pulmonary artery (ALCAPA), occurs in 2 per 100,000 live births and usually presents with heart failure after the postnatal decline in pulmonary resistance. Total anomalous origin of the coronary arteries from the pulmonary artery (TCAPA) is the deadliest coronary malformation, with fewer than 50 cases documented worldwide,^3^ nearly all identified in neonates or at autopsy.^4^ Because both coronaries arise from the low-pressure pulmonary trunk, myocardial perfusion collapses as resistance falls, making survival beyond the neonatal period exceedingly unlikely. We report the first 6-month-old survivor of TCAPA with bilateral intramural course.

## Case Presentation

**Figure 1:**
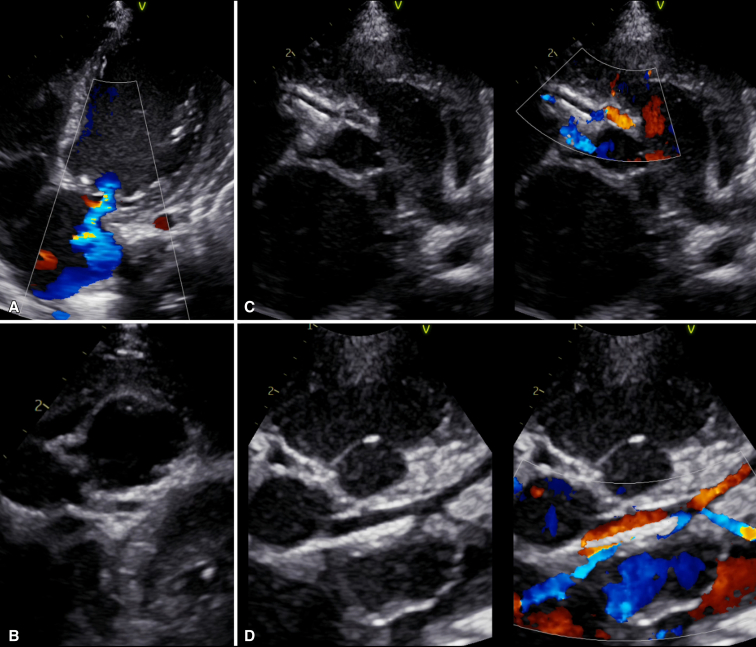
Echocardiographic views demonstrating coronary and valvular anomalies. A, Parasternal 4-chamber view showing ischemic mitral regurgitation. B, Short-axis view falsely suggesting a normal origin of the left coronary artery from the left coronary sinus. C, Short-axis view demonstrating the right coronary artery arising from the pulmonary artery. D, High short-axis view confirming the anomalous origin of both the right and left coronary arteries from the pulmonary artery trunk, with evidence of an intramural course. Unlike anomalous left coronary artery from the pulmonary artery, the left coronary artery flow was observed in red and no collateral vessels or right coronary artery dilatation were identified.

A 6-month-old infant presented to the pediatric emergency department for failure to thrive and tachypnea Over several weeks, he had developed poor feeding endurance, progressive tachypnea with feeds, reduced intake, and weight loss, with weight-for-age declining from +1 to −2 standard deviations. On examination, the infant was tachypneic with a grayish appearance and poor peripheral perfusion (capillary refill time: 3 seconds). Moderate suprasternal and subcostal retractions were noted. Cardiac auscultation revealed a grade 2/6 systolic murmur best heard at the apex, and hepatomegaly was palpable 3 fingerbreadths below the costal margin. Radiography of the chest showed cardiomegaly with mild pulmonary congestion, prompting pediatric cardiology consultation. Electrocardiography demonstrated sinus rhythm at 146 beats per minute with signs of inferolateral ischemia. Laboratory evaluation revealed normal renal and hepatic function but markedly elevated N-terminal pro B-type natriuretic peptide levels (12,000 pg/mL) and mildly increased troponin levels (80 ng/L), consistent with significant myocardial stress. Echocardiography demonstrated a dilated left ventricle with a severely depressed ejection fraction (15%), moderate mitral regurgitation (grade 2/4), and an anomalous origin of the left coronary artery, with strong suspicion of associated right coronary anomaly on the basis of marked interaortico-pulmonary aliasing. Notably, left coronary flow was observed in red, an atypical finding in ALCAPA, and no coronary collateralization or right coronary dilatation was identified (Figure 1). Coronary computed tomography angiography, performed under suboptimal conditions, was inconclusive. Following the French law, institutional review board approval is not required when it comes to anonymous retrospective studies such as case reports. Informed consent was obtained from both parents for publication of the study data.

**Figure 2:**
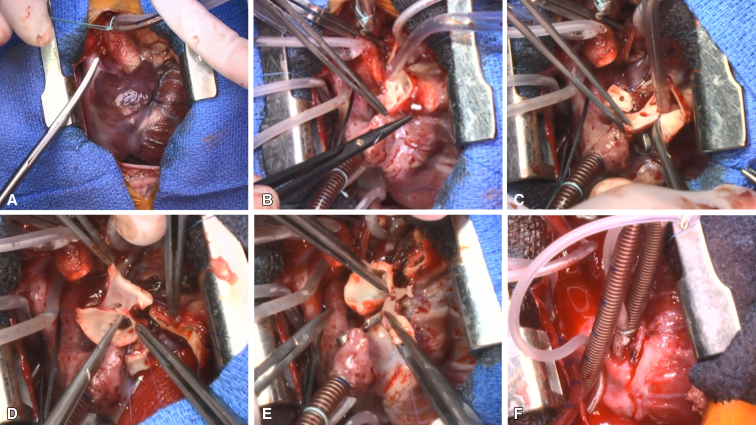
Operative views demonstrating coronary anomalies. A, A median sternotomy was performed, revealing a diffusely violaceous myocardium, suggesting chronic hypoperfusion. B, Intraoperatively, the surgeon confirmed the diagnosis of total anomalous origin of the coronary arteries from the pulmonary artery, noting that both the right coronary artery and left coronary artery originated from the pulmonary artery. C, Coronary buttons were excised, revealing an interaortico-pulmonary and intramural course for both coronaries. D and E, The procedure consisted of bilateral unroofing with neo-ostia creation in the appropriate aortic sinus. F, After the procedure was finished, the myocardium appeared bright red and well perfused, which indicates that it received oxygenated blood through effective revascularization.

**Figure 3:**
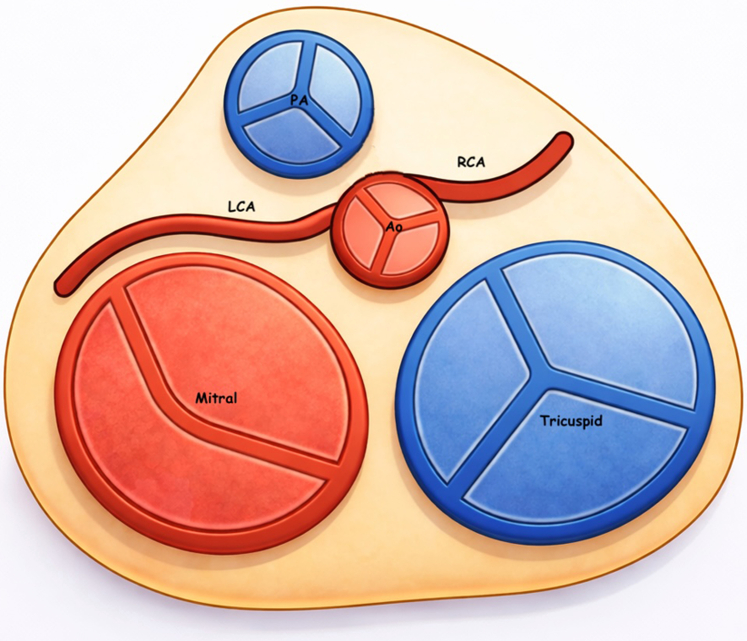
Postoperative aspect. Both vessels emerge from the appropriate aortic sinus. *PA*, Pulmonary artery; *LCA*, left coronary artery; *RCA*, right coronary artery; *Ao*, aorta.

Intraoperatively, the surgeon confirmed the diagnosis of TCAPA, with both right and left coronary arteries originating from the pulmonary trunk and following an interaortico-pulmonary intramural pathway. A median sternotomy was performed, revealing a diffusely cyanotic myocardium, which is suggestive of chronic hypoperfusion. Cardiopulmonary bypass under normothermia was established via aortic and bicaval cannulation. Because the diagnosis was still uncertain, myocardial arrest was induced by administering warm blood cardioplegia in the aortic root as well as in the PA. The main PA was transected, allowing direct visualization of 2 separate coronary ostia, both of which arose from separate sinuses at the posterior aspect of the PA. The coronary buttons were excised. Dissection of the proximal segments of both vessels revealed an interaortico-pulmonary and intramural course for both coronaries. A vertical incision was made in the left sinus, directed toward the intramural portion of the left coronary artery (LCA). The most proximal epicardial portion of the LCA was incised inferiorly and longitudinally until reaching the intramural portion. The 2 incisions were joined and approximated where the intramural portion of the coronary artery became extramural. The healthy proximal segment of the LCA was then used to enlarge and close the aortocoronary incision, therefore creating the neo-ostium in the left sinus. The same technique was repeated for the right coronary artery. Continuity of the great vessels was restored, using a heterologous pericardial patch for the PA. At the conclusion of the procedure, the myocardium appeared bright red and well perfused, indicating effective revascularization with oxygenated blood (Figures 2 and 3 and Video 1). Postoperative recovery was prolonged, with chest closure on day 6, full weaning from vasopressors and mechanical ventilation by day 25, and discharge at 2 months with left ventricular ejection fraction improved to 40% and mild residual mitral regurgitation.

## Discussion

Congenital coronary anomalies remain challenging to diagnose, particularly when clinical signs are subtle. To date, TCAPA with bilateral intramural anomalous origins has not been reported. This exceptional case suggests a compensatory mechanism that allowed survival beyond early infancy without immediate catastrophic decompensation. Unlike ALCAPA, in which myocardial perfusion becomes dependent on collateral flow from the right coronary artery after early infarction, TCAPA precludes the development of collateralization, because the entire coronary circulation originates from the PA. Consequently, myocardial perfusion depends exclusively on PA pressure and oxygen content. In our patient, the bilateral intramural course likely functioned as a restrictive segment, limiting coronary runoff into the PA and delaying ischemic decompensation, as illustrated by the absence of collateral vessels and the atypical antegrade (“red”) coronary flow observed on echocardiography. From a surgical standpoint, the goal of TCAPA repair is to establish reliable aortic-derived coronary circulation. Because of the intramural course of the coronaries, we performed bilateral unroofing to relocate the functional orifice to the appropriate sinus and enlarge it considerably. This strategy enables complete anatomical correction, avoids residual coronary compression or ostial compromise, and provides a durable surgical solution in an otherwise-lethal condition.

## Conclusions

This case illustrates how specific anatomic features can modify the clinical course of TCAPA and demonstrates that complete anatomical repair can be achieved with favorable early outcomes, even in this exceptionally severe coronary anomaly.

## Conflict of Interest Statement

The authors reported no conflicts of interest.

The *Journal* policy requires editors and reviewers to disclose conflicts of interest and to decline handling or reviewing manuscripts for which they may have a conflict of interest. The editors and reviewers of this article have no conflicts of interest.

## Supplementary Data

Online Data Supplement

Video 1 Operative views demonstrating coronary anomalies and repair. Video available at: https://www.jtcvs.org/article/S2666-2507(26)00137-9/fulltext.
