# Supplementary material for: Surgical management of a 6-month-old infant with bilateral intramural total anomalous coronary artery origin from the pulmonary artery
Source: JTCVS Tech. 2026 Mar 20;37:102330. doi: 10.1016/j.xjtc.2026.102330 (PMC13261219; doi:10.1016/j.xjtc.2026.102330)
Supplement: Online Data Supplement [file mmc1.docx]

**Surgical Management of a 6-month-old Infant with Bilateral Intramural Total Anomalous Coronary Artery Origin from the Pulmonary Artery**

**SUPPLEMENTAL MATERIAL**

00’00-00’27: A median sternotomy was performed and cardiopulmonary bypass under normothermia was established via aortic and bicaval cannulation. Myocardial protection was achieved through repeated administration of warm blood cardioplegia.

00’28-00’54: The main PA and aorta were transected, allowing direct visualization of two separate coronary ostia, both of which arose from separate sinuses at the posterior aspect of the PA.

00’55-01’31: The coronary buttons were excised. Dissection of the proximal segments of both vessels revealed an interaortico-pulmonary and intramural course for both coronaries.

01’32-01’43: A vertical incision was made in the left sinus, directed toward the intramural portion of the LCA.

01’44-02’00: The most proximal epicardial portion of the LCA was incised inferiorly and longitudinally until reaching the intramural portion.

02’01-02’08: The two incisions were joined and approximated where the intramural portion of the coronary artery became extramural.

02’09-03’18: The healthy proximal segment of the LCA was then used to enlarge and close the aortocoronary incision, therefore creating the neo-ostium in the left sinus.

03’19-03’24: The most proximal epicardial portion of the RCA was incised inferiorly and longitudinally until reaching the intramural portion.

03’25-03’30: A vertical incision was made in the right sinus, directed toward the intramural portion of the RCA.

03’31-03’39: The two incisions were joined and approximated where the intramural portion of the coronary artery became extramural.

03’40-04’33: The healthy proximal segment of the RCA was then used to enlarge and close the aortocoronary incision, therefore creating the neo-ostium in the right sinus.

04’34-05’45: Continuity of the great vessels was restored, using a heterologous pericardial patch for the PA.

05’46-05’57: At the conclusion of the procedure, the myocardium appeared bright red and well perfused, indicating effective revascularization with oxygenated blood.
