# Supplementary figures and images for: Surgical management of a 6-month-old infant with bilateral intramural total anomalous coronary artery origin from the pulmonary artery
Source: JTCVS Tech. 2026 Mar 20;37:102330. doi: 10.1016/j.xjtc.2026.102330 (PMC13261219; doi:10.1016/j.xjtc.2026.102330)

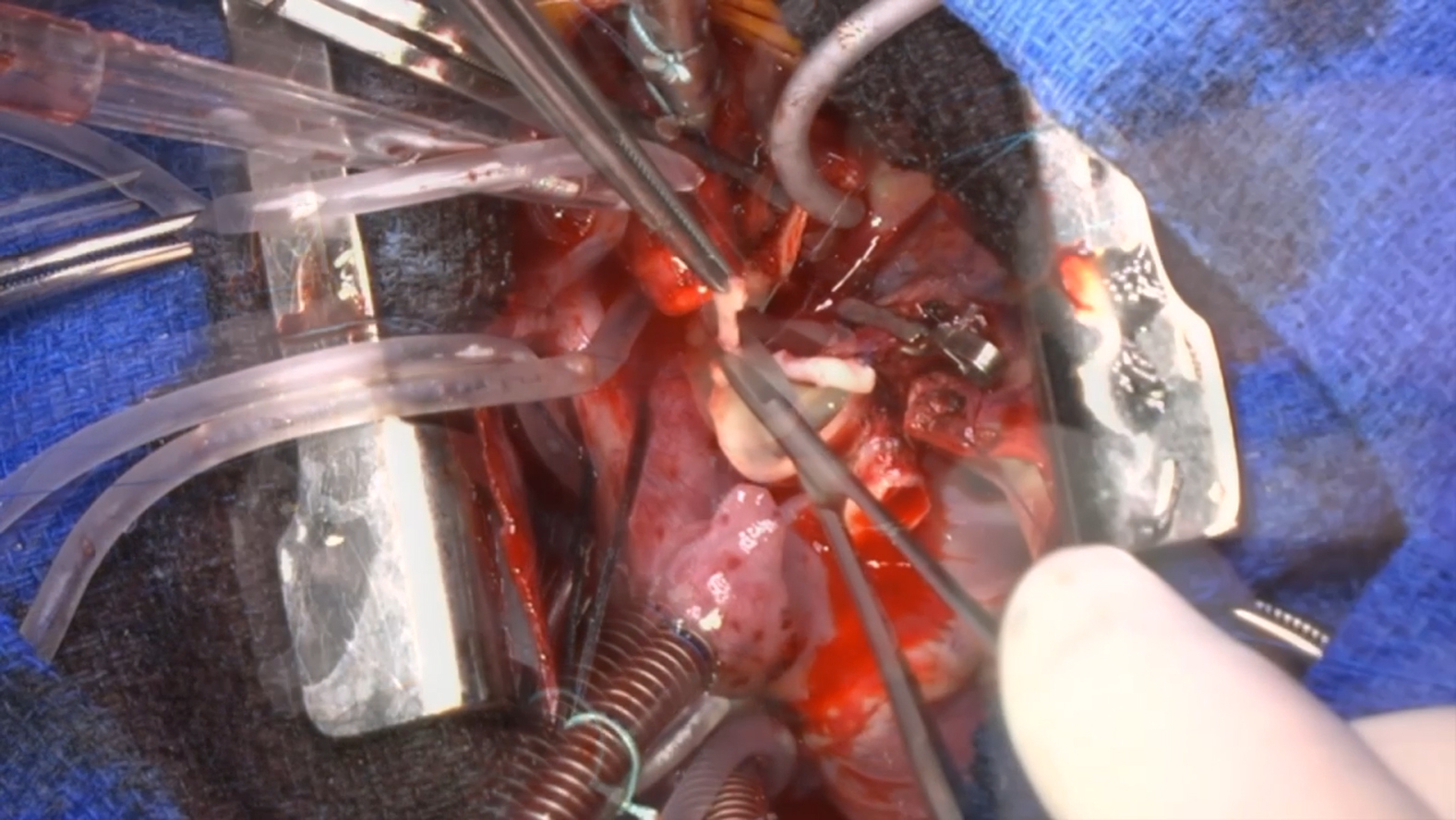

Supplement: Video 1 — Operative views demonstrating coronary anomalies and repair. Video available at: https://www.jtcvs.org/article/S2666-2507(26)00137-9/fulltext. [file fx2.jpg]
